# Partial Depletion of Natural CD4^+^CD25^+^ Regulatory T Cells with Anti-CD25 Antibody Does Not Alter the Course of Acute Influenza A Virus Infection

**DOI:** 10.1371/journal.pone.0027849

**Published:** 2011-11-18

**Authors:** Richard J. Betts, Adrian W. S. Ho, David M. Kemeny

**Affiliations:** Department of Microbiology, Immunology Programme, National University of Singapore, Singapore, Singapore; New York University, United States of America

## Abstract

Foxp3^+^ CD4^+^ regulatory T cells represent a T cell subset with well-characterized immunosuppressive effects during immune homeostasis and chronic infections, and there is emerging evidence to suggest these cells temper pulmonary inflammation in response to acute viral infection. Recent studies have demonstrated treatment with PC61 CD25-depleting antibody potentiates inflammation in a murine model of RSV infection, while paradoxically delaying recruitment of CD8^+^ T cells to the site of inflammation. The present study therefore sought to examine the role of these cells in a murine model of acute influenza A virus infection through the administration of PC61 CD25-depleting antibody. PC61 antibody is able to partially deplete CD25^+^Foxp3^+^ regulatory T cells to a comparable degree as seen within previous work examining RSV, however this does not alter influenza A-virus induced mortality, weight loss, viral clearance and cellularity within the lung. Collectively, these data demonstrate that partial depletion of CD4^+^CD25^+^ regulatory T cells with PC61 antibody does not alter the course of influenza A virus infection.

## Introduction

Regulatory T (Treg) cells are a subset of T lymphocytes capable of moderating inflammatory responses to both foreign and self-antigens, and thus represent a principal mechanism of immune suppression. A diverse assortment of Treg cells have been described in both CD4^+^ and CD8^+^ T cell subsets, however to date most Treg-mediated suppression within mice involves Treg cells expressing the CD4 co-receptor and characteristic transcription factor Foxp3. Within the CD4^+^Foxp3^+^ Treg cells there are believed to be two major subset of Treg cells; natural Treg cells, derived from high-avidity selection for self-antigens within the thymus, and induced Treg cells, which are generated in the periphery from CD4^+^Foxp3^-^ precursors during the course of inflammation [1], [2], [3]. Depletion of Treg cells using α-CD25 antibody PC61 represents a common method of depleting CD25^+^ regulatory T cells, albeit with the potential complication of depletion of natural killer (NK) cells, B cells and effector CD4^+^ T cells bearing CD25 [4].

While there is a growing body of evidence to implicate adaptive Foxp3^+^ Treg cells in tumor resistance to effector responses and various chronic inflammatory conditions including chronic viral infection [5], [6], little is known about the function of Foxp3^+^ Treg cells within acute viral infections, and the role of Treg cells in respiratory viral infection remains poorly defined. A recent series of reports have examined the role of Treg cells in acute respiratory syncytial virus infection, a respiratory virus that induces a mixed Th1/Th2 response during infection [7]. Depletion of Treg cells using PC61 Treg-depleting antibody within RSV infection results in impaired recruitment of antigens-specific CD8^+^ T cells to the lung, while reducing the MHC class I immunodominance hierarchy between the dominant K^d^-restricted M2 epitope towards the sub-dominant D^b^M_187-195_ epitope [8], [9]. While Treg-depleted mice exhibit delayed CD8^+^ T cell infiltration kinetics, responding CD8^+^ T cells produce higher levels of pro-inflammatory cytokines and persist longer in the lung following infection. These data suggest that natural Treg cells assist with the co-ordination of the initial adaptive immune response, but also attenuate inflammation towards the latter stages of infection. Depletion of natural Tregs also results in potentiated innate immunity to RSV, characterized by increased BAL cellularity and elevated cytoktine and chemokine production [10].

While depletion of Treg cells using CD25-depleting antibody results in increased CD8^+^ T cell proliferation, IFN-γ production and cytolytic activity in response to influenza antigens within a murine model of chronic inflammatory bowel disease [11], to date there are very few studies examining regulatory T cells within influenza infection. Longhi and colleagues examined the ability of Treg cells from the spleens of influenza-infected animals to suppress antigen-specific CD4^+^ proliferation at later timepoints, and suggest that IL-6 acts to inhibit the priming of antigen-specific Tregs thus allowing an unconstrained primary CD8^+^ T cell response [12]. Antunes and co-workers noted that adoptive transfer of polyclonal Treg cells into influenza-infected, lymphocyte-deficient mice prolongs survival and attenuates the innate response, demonstrating that Treg cells are capable of altering influenza-induced immunity at least under some circumstances [13]. The present study therefore sought to determine the role of regulatory T cells on the course of influenza A virus infection through the use of PC61 antibody. We find that influenza A virus infection results in the robust induction of a CD4^+^Foxp3^+^CD25^+^ regulatory T cell response. While PC61 CD25-antibody is moderately successful at depleting Treg cells, there is no alteration to clinical signs, viral load or inflammation during infection. These indicate that partial depletion of Treg cells using PC61 antibody does not alter influenza A-virus induced inflammation.

## Results

### Influenza A virus-induced regulatory T cells express high levels of CD25

**Figure 1:**
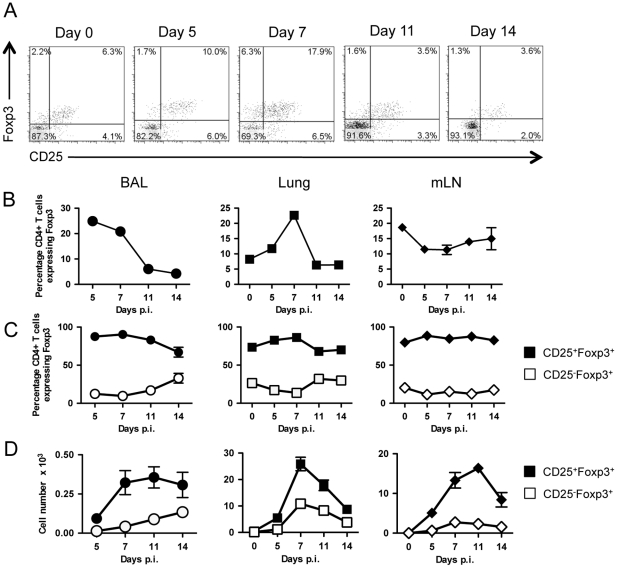
Influenza A virus infection results in the robust induction of a CD25^+^Foxp3^+^ Treg response. Mice were inoculated with 10pfu influenza A/PR8/34 and sacrificed at various timepoints and the induction of CD25^+^Foxp3^+^ Treg cells determined across the course of infection. (A) Representative FACS plots of the proportion of T cells collected from BAL expressing CD25 and Foxp3, gated on CD3 and CD4 positive cells. (B) Proportion of CD3^+^CD4^+^ T cells expressing Foxp3^+^ across a timecourse in cells collected from BAL, lung, and mediastinal lymph node. (C) Proportion of Foxp3^+^ Tregs that co-express CD25^+^ across a timecourse in cells collected from BAL, lung, and mediastinal lymph node. (D) Total numbers of CD25^+^Foxp3^+^ and CD25-Foxp3^+^ T cells across a timecourse in cells collected from BAL, lung, and mediastinal lymph node. Data represents mean ± S.E.M, n = 4–7 for all.

As the present study sought to eliminate regulatory T cells using α-CD25 (PC61) antibody, it was necessary to examine the expression of CD25 on influenza A virus-induced Treg cells. At the site of inflammation there is a preferential recruitment of Treg cells at early timepoints, with the peak proportion of CD4^+^ T cells expressing Foxp3 being at day 7 within the lung, while the proportion of Treg cells falls within the draining lymph node at day 7 (Fig. 1B). Initially, only ∼75% of Treg cells within the lung express CD25, increasing to ∼90% by day 7 post-inoculation. In both BAL and lung the proportion of Foxp3^+^ expressing CD25 remain elevated at day 11 before decreasing at day 14 post-inoculation (Fig. 1C). These findings suggest Treg cells induced within influenza will be susceptible to α-CD25 PC61-mediated depletion, but that a sizeable proportion of Foxp3^+^CD25^-^ cells may remain and represent a possible confounding factor.

### Influenza A virus-induced Treg cells are partially depleted by PC61 antibody

**Figure 2:**
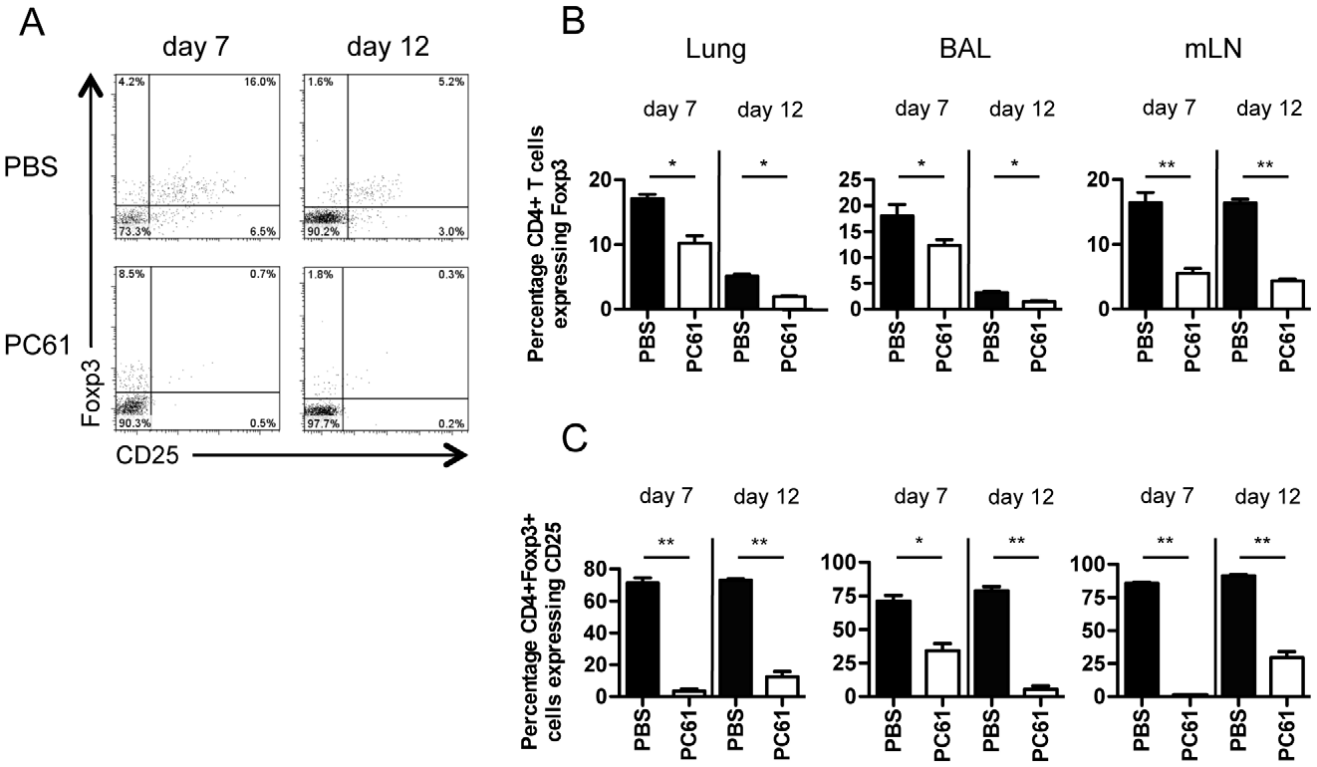
PC61 α-CD25 antibody administration results in partial depletion of Foxp3^+^ regulatory T cells. Mice were inoculated with 10pfu influenza A/PR8/34 and administered PBS or 400 µg of PC61 antibody i.v. at days -2 and 4 post-inoculation, and expression of CD25^+^ and Foxp3^+^ within CD3^+^CD4^+^ T cells examined at days 7 and 12 post-inoculation within lung, BAL and mLN samples. (A) Representative FACS plots of CD25 and Foxp3 expression on BAL CD3^+^CD4^+^ T cells at days 7 and 12 post-inoculation in PC61 and PBS-injected mice. (B) Percentage of CD4^+^ T cells expressing Foxp3^+^ in cells collected from lung, BAL and mLN of PBS and PC61 treated mice at days 7 and 12 post-inoculation. (C) Percentage of CD4^+^Fopx3^+^ T cells co-expressing CD25 in PBS and PC61 treated mice from lung, BAL and mLN of PBS and PC61 treated mice at days 7 and 12 post-inoculation. * denotes t<0.05, ** t<0.01, Student' t-test. Data represents mean ± S.E.M, n = 6–8 for all.

We next administered mice PC61 CD25-depleting antibody to influenza A virus-infected mice twice, at days -2 and 4 post-inoculation via the tail vein, and the mice were sacrificed at day 7 or day 12 post-inoculation. A dual dose protocol was used as preliminary experiments revealed a single injection of PC61 was ineffective as reducing Treg numbers by day 7 post-inoculation (data not shown). At day 7 post-inoculation lung and BAL Foxp3^+^ Treg numbers has only been modestly reduced by ∼30%, in contrast to the ∼65% depletion observed within the draining lymph node (Fig. 2B). Depletion was more extensive within the lung and BAL by day 12 post-inoculation, with ∼60% Treg reduction.

Few Foxp3^+^ Treg cells within the lung and mLN stained positive for CD25 at day 7 post-inoculation, suggesting extensive binding of PC61 antibody to target CD25 (Fig. 2C). Approximately 40% of Treg cells within the BAL stained positive for CD25 however, demonstrating that PC61 antibody may not be penetrating the BAL compartment efficiently at early timepoints which may account for the inefficient BAL Treg depletion at day 7 post-inoculation (Fig. 2C). Foxp3^+^CD25^+^ proportions had begun to recover by day 12 however, presumably as circulating antibody levels are reduced. BAL Foxp3^+^CD25^+^ proportions were reduced however, possibly a reflection of the reduced amount of Treg cells expressing CD25 at the latter stages of infection. Collectively these results demonstrate PC61 administration results in a moderate degree of Treg depletion within influenza infection, and is more effective at later timepoints.

### Partial depletion of Treg cells does not alter influenza A-virus induced weight loss or mortality

**Figure 3:**
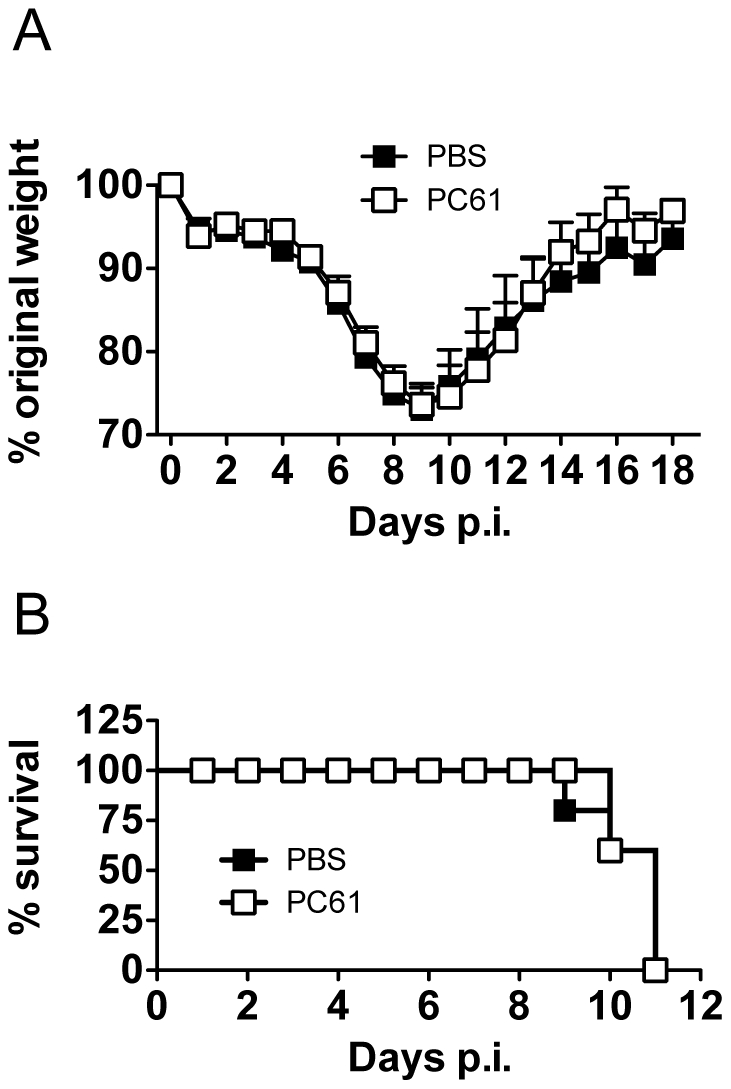
PC61 α-CD25 antibody administration does not alter influenza A virus-induced weight loss of influenza A-virus induced mortality. Mice were inoculated with influenza A/PR8/34 and administered PBS or 400 µg of PC61 antibody i.v. at days -2 and 4 post-inoculation. (A) Influenza-induced weight loss in mice inoculated with a sub-lethal dose of 10pfu influenza A virus and administered PBS or 400 µg PC61 α-CD25 antibody. (B) Influenza A virus-induced mortality in mice inoculated with lethal dose of 250pfu Influenza A virus and administered PBS or 400 µg PC61 α-CD25 antibody. Data represents mean ± S.E.M, n = 6 for all.

Having established PC61 antibody administration results in a reduction of Treg numbers, we sought to determine if depletion of Treg cells alters influenza A-virus induced weight loss and mortality. Sublethal infection influenza A virus infection resulted in progressive weight loss up to day 9 post-inoculation, after which the mice had recovered by day 18 post-inoculation (Fig. 3A). Administration of PC61 antibody did not alter influenza A virus-induced weight loss within sub-lethal infection (Fig. 3A). At a lethal dose of 250pfu/mouse, PC61 administration similarly did not alter influenza A virus-induced mortality (Fig. 3B).

### Partial depletion of regulatory T cells does not alter influenza A virus-induced pulmonary inflammation

**Figure 4:**
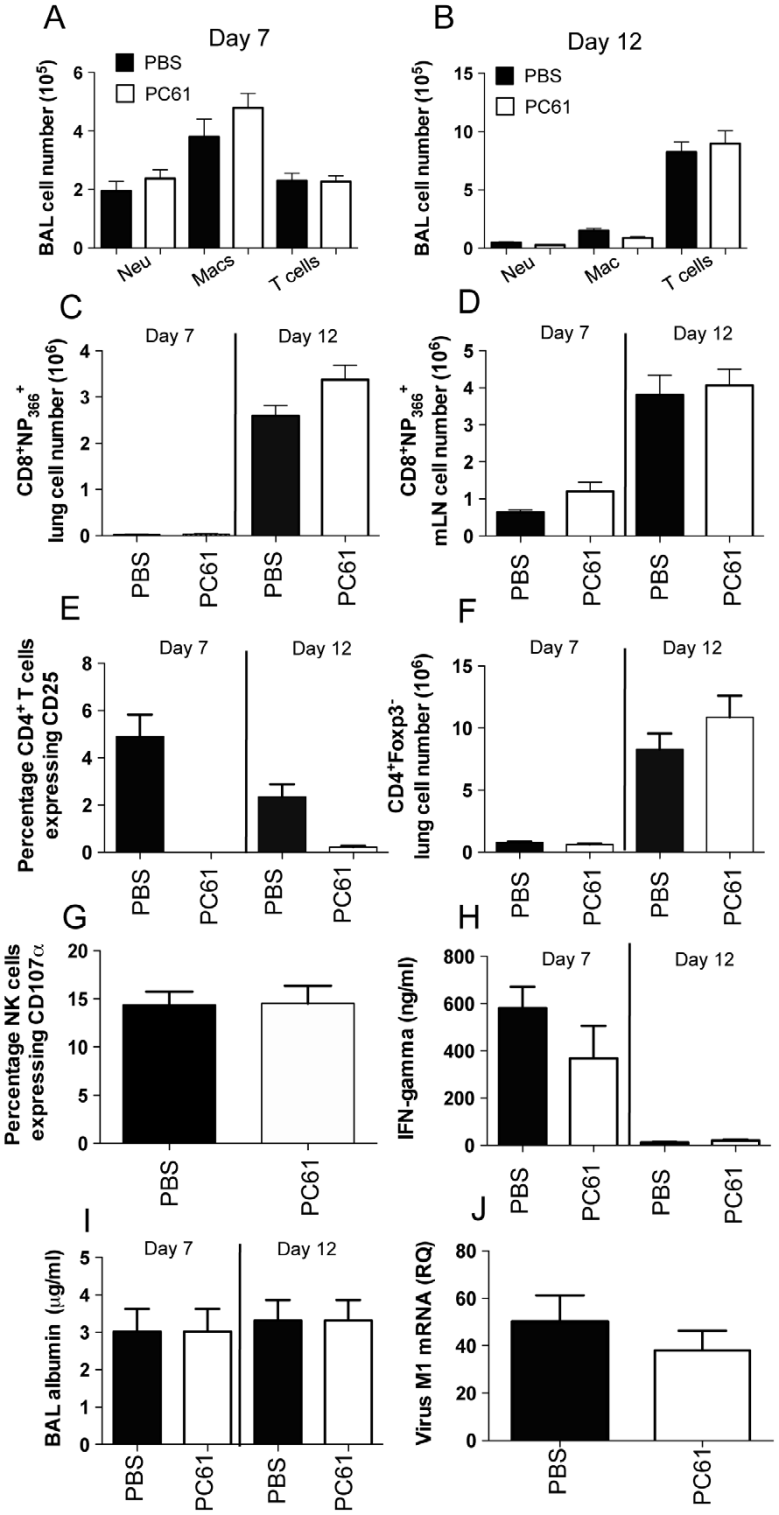
PC61 α-CD25 antibody administration does not alter influenza A virus-induced BAL cellularity, antigen-specific CD8^+^ T cell numbers, CD4^+^Foxp3^-^ T cell numbers, NK cell activation, IFN-γ levels, immunopathology and pulmonary viral load. alter influenza A virus-induced weight loss of influenza A-virus induced mortality. Mice were inoculated with influenza A/PR8/34 and administered PBS or 400 µg of PC61 antibody i.v. at days -2 and 4 post-inoculation. (A) BAL cellularity as determined by flow cytometry at day 7 (A) or day 12 (B) post-inoculation in mice administered PBS or PC61 α-CD25 antibody. Antigen-specific CD8^+^ T cell numbers in lung (C) or mLN (D) at days 7 and 12 post-inoculation in mice administered PBS or PC61 antibody. (E) Percentage of CD4^+^Foxp3^-^ T cells expressing CD25 and (F) total CD4^+^Foxp3^-^ T cell numbers at days 7 and 12 post-inoculation in mice treated with PBS or PC61 antibody. (G) Percentage NK cells expressing activation marker CD107α at day 2 post-inoculation in mice administered 400 µg of PC61 antibody i.v. at days -2 post-inoculation. (H) IFN-γ levels in BAL fluid at days 7 and 12 post-inoculation in mice administered PBS or 400 µg of PC61 antibody i.v. at days -2 and 4 post-inoculation. (I) BAL albumin at days 7 and 12 post-inoculation in mice administered PBS or 400 µg of PC61 antibody. (J) Pulmonary viral titre at day 7 post-inoculation in mice administered PBS or PC61 antibody. Data represents mean ± S.E.M, n = 5–7 for all.

Previous studies examining the role of Treg cells within RSV have noted potentiated pulmonary inflammation with the BAL fluid of Treg-depleted mice. We therefore treated influenza A virus-inoculated mice with PC61 antibody and examined BAL cellularity using flow cytometry at days 7 and 12 post-inoculation, to coincide with the timepoints at which Treg infiltration is proportionally at its greatest and with the peak of adaptive immune response, respectively. Surprisingly, treatment with PC61 antibody did not alter pulmonary infiltration of neutrophils, macrophages and lymphocytes at both time-points examined (Fig. 4A & B). As depletion of regulatory T cells has been shown to alter antigen-specific CD8^+^ T cell infiltration, we examined the numbers of influenza antigen-specific CD8^+^ T cells specific for immunodominant Class I epitope NP_366_ into the lung and draining lymph node at days 7 and 12 post-inoculation. Administration of PC61 did not alter the number of NP366^+^CD8^+^ Treg cells within either the draining lymph node or lung at both time-points examined (Fig. 4C & D). While PC61 antibody bound to target CD25 on the minority CD25^+^ population of CD4^+^Foxp3^+^ T cells at both timepoints examined (Fig. 4E), total numbers of CD4^+^Foxp3^-^ remained unchanged (Fig. 4F). PC61 administration did not alter NK cell activation as determined by CD107α expression at day 2 post-inoculation (data not shown). We also examined BAL levels of characteristic Th1 cytokine IFN-γ, which is produced in high levels within influenza infection [14]. IFN-γ levels were unchanged by PC61 administration (Fig. 4G), as were BAL serum albumin levels, a characteristic marker of pulmonary immunopathology [15] (Fig. 4H). Viral clearance, as measured by pulmonary viral load at day 7 post-inoculation, was similarly unchanged (Fig. 4I). Collectively these results indicate that administration of PC61 depleting antibody does not alter the inflammatory response to influenza A virus infection.

## Discussion

Influenza A virus infection results in a vigorous immune response, with extensive early innate immunity characterized by neutrophilia, leading to a robust adaptive immune response in which large numbers of CD8^+^ T cells infiltrate the site of infection. While CD8^+^ T cells are protective against illness and lung injury at low pulmonary viral loads [16], at high viral loads they cause a high degree of immunopathology that may lead to significant morbidity and mortality [17]. With such virus-induced immune dysregulation a prominent feature of the disease, we postulated regulatory mechanisms such as the induction of a Treg response during the course of influenza infection would be vital in balancing the immune response to the virus. Within the present study however we find no evidence that partial Foxp3^+^ Treg cells contribute to the pathogenesis of influenza A virus infection.

Our findings of the proportions of Foxp3^+^ Treg cells expressing CD25^+^ are broadly consistent with those reported for RSV infection [8], [9], [10], demonstrating that Treg cells induced during influenza A virus infection are a viable target for depletion via a CD25-depleting antibody. Despite using a higher dose of PC61 antibody than Lee and colleagues and a comparable dose to Ruckwardt and co-workers however, we find Treg depletion to be slightly less efficient within influenza A virus infection compared to RSV, and similar to the case for RSV infection there remains a substantial pool of Foxp3^+^CD25^-^ Treg cells within all tissues examined which represents a considerable confounding factor. CD25 expression on Foxp3^+^ cells is highly plastic [18], [19], and CD25 is not an absolute requirement for suppressive ability [20]. Foxp3^+^CD25^-^ Treg cells that persist after depletion possess potent regulatory function [21], and are capable of rapidly upregulating CD25 following PC61 administration [22]. This incomplete depletion therefore raises the possibility of underestimation of the role Foxp3^+^ Treg cells play within influenza infection.

Within the present study, depletion of Treg cells using PC61 antibody does not alter influenza-induced weight loss, pulmonary viral clearance, immunopathology or lung cellularity. These findings are surprising in light of previous studies utilizing similar Treg depletion protocols within a murine model of RSV infection, variously noting reduced viral loads, increase virus-induced weight loss, potentiated inflammation and altered antigen-specific CD8^+^ T cell kinetics [8], [9], [10]. Within murine models of RSV infection there is comparatively limited viral replication and correspondingly mild lung pathology [23], and within our hands RSV infection results in a considerable milder disease state compared to the high pathogenicity induced by the PR8 strain of influenza in mice. A cross-comparison of BAL and lung cell counts obtained within these RSV studies with those of our influenza study reveals a stronger inflammation generated by Influenza A/PR8, with a corresponding higher degree of virus-induced weight loss. As even a small dose of influenza virus is capable of inducing a strong inflammatory response within mice [24], the higher intrinsic pathogenicity of influenza A virus may account of the attenuated efficacy of PC61. From these results one can draw two possible conclusions; that CD25^+^Foxp3^+^ Treg cells are not an influential factor in the inflammatory response to influenza A virus, or that the technical limitations of the PC61 CD25-depletion approach results in insufficient Treg depletion, with Treg cell numbers not being reduced the level required to observe effects. Previous authors demonstrating comparable levels of Treg depletion to those observed within the present study have frequently concluded the former, that lack of efficacy of PC61 demonstrates the relative unimportance of Treg cells within the relevant model of infectious disease [25], [26], [27]. A recent study conducted by Li and co-workers however has demonstrated that partial depletion of Treg cells through PC61 does not alter tumor size within a murine inoculated with M04 melanoma, but that 95% ablation through the use of Foxp3-DTR transgenic animals results in complete regression of large established tumors and an associated increase in tumor-specific CD8^+^ T cell infiltration [28]. These findings raise the possibility that Treg cells are of greater importance within infectious disease than previous thought, but that technical limitations have precluded the precise examination of their contribution to resolving infection. We therefore are unable to completely rule out a role of Treg cells within influenza, but note that reduction of Treg cells to the levels achievable through PC61 depletion, and comparable to those observed within RSV models, does not alter disease outcome. The findings by Antunes and colleagues that adoptive transfer of Treg cells into lymphocyte-deficient mice suggests that Treg cells are capable of modulating influenza immune responses to some degree [13], however there remains the possibility that the role of Treg cells within influenza is dependent on experimental conditions.

In conclusion, within the present study we demonstrate that administration of with PC61 α-CD25 results in partial depletion of influenza A virus-induced Foxp3^+^ Treg cells, but that despite positive results obtained within other murine models of acute respiratory viral infection this reduction in Treg cell numbers does not alter the course of the disease. While we find no support for the hypothesis that Treg cells are able to alter influenza A virus infection, previous research examining regulatory T cells within influenza and emerging data concerning the relative lack of efficacy of PC61 antibody suggests these results should be interpreted with caution.

## Materials and Methods

### Mice and virus stocks

C57BL/6 mice were purchased from Jackson Laboratories and bred in-house at the National University of Singapore animal facility. Mice were age and sex-matched for each experiment. All experiments were carried out in accordance of the guidelines of The National University of Singapore Advisory Committee for Laboratory Animal Research (NACLAR) Singapore. The protocols (protocol numbers 094/06 and 137/08) were approved by Institutional Animal Care and Use Committee (IACUC) of the National University of Singapore. Influenza A/PR/8/34 (H1N1) was purchased from ATCC and propagated in the allantois of 10 day-old embryonated chicken eggs and stored at −80°C.

### Mouse infection and treatment

Intranasal challenge of mice with influenza was performed by injecting mice with ketamine (100 mg/kg, Sigma) and medotomidine (15 mg/kg, Orion Pharma) i.p. and administering 10 pfu of influenza in 20 µl volume i.n., after which mice were administered atipamezole (5 mg/kg, Pfizer) reversal. In mortality experiments mice were administered 250 pfu influenza A virus in 20ul. PC61 α-CD25 antibody was generated from hybridoma cells (ATCC) in serum-free media (Gibco), and purified using HPLC. For Treg depletion, mice were administered 400 µg of PC61 antibody in PBS i.v. at days -2 and 4 post-inoculation. All animal experiments were conducted in accordance with institutional guidelines.

### T cell collection

Spleen, mediastinal lymph node (mLN) and mesenteric and axillary non-draining lymph nodes (NDLN) were collected from euthanized animals and pushed through a 61 µm cell strainer (BD Falcon) to obtain a single cell suspension. BAL samples were collected by 4×0.5 ml instillations of PBS through the trachea of cannulated mice. Lung samples were digested for 45 minutes in Liberase (Roche) at 37°C prior to being passed through a cell strainer. Red blood cells were lysed using ammonium chloride. Cell samples were washed with MACS buffer and stained for surface markers CD3 (Clone 145-2C11, Biolegend) and CD4 (clone RM4-5, BD Pharmingen) and CD25 (Clone PC61, Biolegend) for 30 mins. Cells were washed twice, fixed using fixation/permeabilization buffer (BD Bioscience) for 1 hour, washed twice in permeabilization buffer and stained for Foxp3 (Clone FKJ-16s, eBioscience) for 30 mins. Cells were washed twice and examined on a CyAn flow cytometer (Beckman Coulter). Antigen-specific CD8^+^ T cell numbers were determined using NP_366_ tetramer (Proimmune). NK cell activation status was determined by activating ficoll-enriched lung cells with PMA (50 ηg/ml)/Ionomycin (400ηg/ml) in the presence of α-CD107α antibody, monensin (1 µl/ml) and brefeldin A (1 µl/ml) for 3.5 hours, after which they were stained for CD3, NK1.1 and CD25.

### BAL cellularity

BAL cellularity was determined by flow cytometry. In brief, BAL samples obtained as described above, washed in MACS buffer and stained for 30 mins within CD3 (T cells, Clone 145-2C11, Biolegend), Ly6G (neutrophils, Clone EL-4J, Biolegends) or CD11c (macrophages, Clone M5/114.15.2, Biolegend).

### Cytokine and BAL Albumin ELISA

Cytokine levels were examined within BAL fluid by ELISA (R&D Systems) according to manufacturers instruction, while BAL albumin levels were determined using serum albumin ELISA kit (Bethyl Laboratories). In brief, Immunosorb ELISA plates (Nunc) were coated overnight with capture antibody, washed and blocked with 1% BSA at room temperature for 1 hour. Samples or standard were added for 2 hours, washed, and bound antibody was detected using biotinylated anti-cytokine antibody, avidin horseradish peroxidase and tetramethylbenzidine. Color development was halted with 2M H_2_SO_4_ and optical density read at 450 nm.

### Quantification of viral RNA

Mice were sacrificed and the left lung lobe removed and homogenized within RNA later (Qiagen). Viral load was determined using real-time quantitative PCR and influenza matrix gene M1 as described elsewhere [29]. Data is expressed as a percentage of GAPDH output.

### Statistical analysis

Statistic analysis was performed using GraphPad Prism (GraphPad Software). Pairwise comparisons were made using Student's t-test, while comparisons across groups were made using 1-way ANOVA and dual comparisons determined using student's t test.
